# Progress in Dermatology

**Published:** 1900-07-21

**Authors:** 


### Progress in Dermatology.

(*Continued from page* 256.)

**Alopecia.**—Brown[18] reports two interesting epidemics of alopecia occurring in an institution for orphan girls. Several months after a young girl had been attacked with baldness the disease broke out among the other inmates, 63 out of 69 being attacked. After several years' absence the original patient returned to the asylum and again gave rise to an epidemic, 26 out of 45 inmates becoming affected. The disease differed from ordinary alopecia only on account of the smallness of the patches affected, but numerous examinations of the hair failed to reveal any parasites.

Eddowes[19] describes the case of a male, aged 16, with almost complete alopecia. After treatment by thyroid extract internally, and lysol externally, the hair was rapidly returning. He considered the case to be one of leucoderma of the hairy parts.

Walker[20] puts the remedies for the treatment of alopecia in the following order: (1) sulphur; (2) chrysorobin (this may be applied either dissolved in chloroform or in the form of a stick); (3) perchloride of mercury in spirit of the strength of 1 or ½ per cent. Other remedies are turpentine, paraffin oil, or ammonia lotion.

Jersild[21] has employed Finsen's concentrated chemical rays in cases of alopecia. The main points are (1) the bactericidal action of light and its penetrating power; (2) the favourable action of light on the growth of hair; (3) the provocation of local irritation. The treatment lasted from one to four weeks, with from 4 to 48 sittings of 1 hour each. In all cases the growth of hair was very rapid, and the hairs were firm and non-pigmented. In one case the hair did not appear until after one month's treatment.

**Leprosy.**—Lewers[22] reports a case of leprous fever in a patient at the Charing Cross Hospital, and discusses the frequency and nature of febrile attacks in leprosy. Although these attacks have been confounded with malaria, they bear a closer resemblance to the fever of phthisis. He arrives at the following conclusions: (1) That there is an essential leprous fever due to the presence of the bacillus lepræ or its toxins; (2) that this fever is always intermittent; (3) it may occur at any period of the disease, including the podronal period; (4) it probably occurs in all forms of the disease, although in varying degrees; (5) fever of a continued type is due to the presence of other toxins; (6) it has little clinical import beyond indicating that the disease is active and progressive.

Thompson[23] reviews the conditions under which leprosy is found in Madeira. The disease is known to have existed for over 400 years; and, though no precautions for isolation are taken and the lepers themselves are not avoided by the healthy inhabitants, the disease is not on the increase, only 70 lepers being found on the island, or an average of 6 in 10,000. This fact, in the author's opinion, militates against the infective character of leprosy.

Carrasquilla[24] reports that he has cultivated the lepra bacillus in human blood-serum. Two forms were observed: (1) Long and slender; (2) short and almost elliptical-shaped bacilli. He maintains that this is identical with Hansen's bacillus, because—(1) It resists decolouration by 30 per cent. nitric acid; (2) no other organism was introduced when the tubes were inoculated; (3) the filtered fluid from cultures produced the same reaction on horses as lepers' blood-serum; (4) the serum from horses, subjected to injections with culture filtrates, produces the same reaction in lepers as the serum from horses infected with leper's blood; and (5) the bacillus stains in the same way as Hansen's bacillus.

Tourtoulis-Bey[25] relates the case of a Copt, in whom the leprous macules appeared some months after an attack of leprous fever at the age of 15. There was no family history, but there were lepers living in his village. He was first seen at the age of 35, when he presented a typical leprous appearance. Thirty to 50 drops of chaulmougra oil were given in milk three times a day with some improvement, but had to be discontinued on account of vomiting. Fresh lepromata then developed, and the oil was again given, this time in capsules; but had to be discontinued for the same cause. Later the oil was given subcutaneously, and improvement was marked after about 50 injections. During six years he was injected 584 times, amounting to a total of 2,720 grammes of chaulmougra oil. The injections were made on the outer sides of the upper and lower limbs with a long needle; they were practically painless, and no abscess occurred. He does not consider that this case proves that subcutaneous injection will cure leprosy on account of the possibility of spontaneous retrogression of lepromata.

Hallopeau[26] mentioned that he had injected chaulmougra into the buttocks, in 10 c.m. doses once a week; but in one case there was a severe outbreak of nodules, which led him to give up this treatment. Du Castel[27] is trying the subcutaneous method at Saint Louis Hospital.

Schmidtmann[28] describes the leper hospital at Jerusalem. This accommodates 140 patients, but many mild cases are at large, and the lepers in the hospital mix freely with the other inhabitants. The doctor, who has been in charge for 33 years, is convinced that no case of direct infection has occurred, and considers that heredity is the important factor in the ætiology of the disease.

Silverman[29] records the case of a man, aged 52, who had an eruption of syphilitic leucoderma which closely resembled anæsthetic macular leprosy. He had suffered from other manifestations of syphilis, and so the diagnosis was easy.

**Therapeutics.** — **Tannaform**: Ehrmann[30] has used tannaform in the treatment of skin disease due to disturbance of secretion, such as hyperidrosis, eczema, and balanitis. It was used as a powder mixed with talc (1 in 4 or 5) for hyperidrosis and balanitis, and as a 25 per cent. ointment with lanolin for eczema. A 5 per cent. ointment is sufficient in eczema intertrigo of children. The best results were obtained in cases of occupation eczema. The ointment was applied on linen, and changed night and morning.

Ullmann[31] has used tannaform in 154 various cases of skin disease. It is applied in the form of a powder or ointment. In hyperidrosis, he dusts on a powder of the strength of 1 in 3. In eczema, the best results are obtained when the disease is the result of hyper-secretion. The only disadvantage of the remedy is the staining of the linen, which can, however, be easily removed by moistening the spot, before washing, with a concentrated solution of ammonium sulphate.

**Cacdylic Acid.**—Gyselman[32] gives the result of the hyperdermic injection of cacdylate of soda in the treatment of psoriasis. This compound is an organic arsenical salt, and has the property of diminishing infiltration and hyperæmia. He does not think that its use is superior to other forms of arsenic in psoriasis, but in lichen planus better results are obtained than with Fowler's solution.

Danlos[33] considers that the great advantage in the use of the cacodylates is that it is possible for a patient to take colossal doses of arsenic without danger. Its disadvantages are the oniony odour of the breath, fetid stools, sometimes colic, and occasionally exfoliative dermatitis. He has found it most useful in psoriasis, lichen, lupus erythematosus, and in Duhring's disease; further, he strongly recommends its employment in cutaneous sarcomata. The dose employed is from ·40 to ·60 grs. for a man and ·30 gr. for a woman. He believes that the drug is perfectly harmless.

**Resorcin.**—Friekenhaus[34] recommends the treatment of seborrhœa by vigorous rubbing, with a 25 per cent. alcoholic solution of resorcin each night. The treatment is painless, and at the end of eight days there is a great and general improvement. Pityriaisis versicolor can be treated in the same way. When the price of the remedy is of importance, salicylic acid can be substituted for resorcin, and a 1 in 6 alcoholic solution employed.

**Sapodermia.**—Bucknell[35] recommends sapodermia in the treatment of parasitic diseases; it is a soap in which bichloride of mercury is incorporated with triple refined stearin and glycerin. The bichloride is, therefore, changed into an albuminate of mercury, which is a very active antiseptic.

**Endermol.**—Wolters[36] has used endermol (nicotine salicylate) in 63 cases of scabies. In no case were more than six applications required. A one per cent. ointment was used; if used in stronger proportions toxic effects may be produced. It is free from odour and does not stain linen.

**Radio-therapeutics.**—Freund[37] has employed the *x* rays therapeutically with great success. Superfluous hairs are removed painlessly, and without inconveniencing the patient. In two months he has cured alopecia; lupus vulgaris and erythematosus have healed, and he has been equally fortunate in sycosis and favus; moreover, he has had good results in chronic eczema, necrotic acne, and boils.

[18] Jour. of Cut. and Genito-Urinary Dis., Sept., 1899; Int. Med. Mag., Dec., 1899, p. 927; Brit. Jour. of Derm., Nov., 1899, p. 439. [19] Lancet, Feb, 10, 1900, p. 386. [20] Clin. Jour., Jan. 7, 1900. [21] Ann. de Derm. et de Syph, Jan., 1899; Brit. Med. Jour., Ep., Oct. 14, 1899, No. 307. [22] Brit. Jour. of Derm., Oct., 1899; Int. Med. Mag., May, 1900, p. 927. [23] Lancet, Sept. 30, 1899, p. 885. [24] Brit. Med. Jour. Ep., Oct. 14, 1899, No. 34. [25] Ditto, Nov. 11, 1899, No. 382; Ann. de Derm. et de Syph., July, 1899. [26] Ditto, ditto. [27] Ditto, ditto. [28] Derm. Zeitch, Nov., 1899, p. 58; Brit. Jour. of Derm., Feb., 1900, p. 76. [29] New York Med. Jour., Nov. 18, 1899, p. 729. [30] Wien. Med. Blatt., No. 46, 1899; Brit. Med. Jour. Ep., Sept. 16, 1899, No. 224. [31] Central Therap., May, 1899; The Therapist, July, 1899, p. 303. [32] Wien. Klin. Woch., No. 14, 1899; Brit. Jour. of Derm., Nov., 1899, p. 448. [33] Des Nouveaux Remed., Sept. 24, 1899, p. 484. [34] Ditto, Nov. 24, 1899, p. 520. [35] New York Med. Jour., Feb. 24, 1900, p. 253. [36] Brit. Med. Jour. Ep., Feb. 16, 1900, No. 223; Ther. Monat., Aug., 1899. [37] Wien. Klin. Woch., 1899, No. 39; Brit. Jour. of Derm., Dec., 1899, p. 482.

